# Cross-cultural invariance of the Spanish version of the COVID-19 Assessment Scorecard to measure the perception of government actions against COVID-19 in Latin America

**DOI:** 10.1186/s41155-023-00277-9

**Published:** 2023-11-08

**Authors:** Tomás Caycho-Rodríguez, Pablo D. Valencia, José Ventura-León, Carlos Carbajal-León, Lindsey W. Vilca, Mario Reyes-Bossio, Mariel Delgado-Campusano, Daniel E. Yupanqui-Lorenzo, Rubí Paredes-Angeles, Claudio Rojas-Jara, Miguel Gallegos, Mauricio Cervigni, Pablo Martino, Roberto Polanco-Carrasco, Diego Alejandro Palacios, Rodrigo Moreta-Herrera, Antonio Samaniego-Pinho, Marlon Elías Lobos Rivera, Andrés Buschiazzo Figares, Diana Ximena Puerta-Cortés, Ibraín Enrique Corrales-Reyes, Raymundo Calderón, Walter L. Arias Gallegos, Olimpia Petzold, Andrés Camargo, Julio Torales, J. Arkangel Monge Blanco, Pedronel González, Vanessa Smith-Castro, Wendy Yamilet Matute Rivera, Daniela Ferrufino-Borja, Paula Ceballos-Vásquez, Agueda Muñoz-del-Carpio-Toia, Jorge Palacios, Carmen Burgos-Videla, Ana María Eduviges Florez León, Ibeth Vergara, Diego Vega, Nicol A. Barria-Asenjo, Marion K. Schulmeyer, Hassell Tatiana Urrutia Rios, Arelly Esther Lira Lira

**Affiliations:** 1 Facultad de Psicología, Universidad Científica del Sur, Campus Villa II, Ctra. Panamericana S 19, Villa EL Salvador, Lima, Peru; 2 Facultad de Estudios Superiores Iztacala, Universidad Nacional Autónoma de Mexico, Tlanepantla de Baz, State of Mexico Mexico; 3 Facultad de Ciencias de la Salud, Universidad Privada del Norte, Lima, Perú; 4 South American Center for Education and Research in Public Health, Universidad Norbert Wiener, Lima, Perú; 5 Facultad de Psicología, Universidad Peruana de Ciencias Aplicadas, Lima, Peru; 6 Escuela de Psicología, Universidad de Ciencias y Humanidades, Lima, Perú; 7 CRONICAS Center of Excellence in Chronic Diseases, Universidad Peruana Cayetano Heredia, Lima, Peru; 8 Facultad de Ciencias de la Salud, Departamento de Psicología, Universidad Católica del Maule, Talca, Chile; 9 Programa de Pós-Graduação em Psicologia, Universidade Católica de Minas Gerais, Belo Horizonte, Brasil; 10 Centro Interdisciplinario de Investigaciones en Ciencias de la Salud y del Comportamiento, Consejo Nacional de Investigaciones Científicas y Técnicas, Entre Ríos, Argentina; 11 Facultad de Psicología, Universidad Nacional de Rosario, Rosario, Argentina; 12 Centro Interdisciplinario de Investigaciones en Ciencias de la Salud y del Comportamiento, Universidad Adventista del Plata, La Plata, Argentina; 13 Laboratorio de Investigaciones en Ciencias del Comportamiento (LICIC), Facultad de Psicología, Universidad Nacional de San Luis, San Luis, Argentina; 14 Cuadernos de Neuropsicología, Rancagua, Chile; 15 Centro de Desarrollo Humano, Universidad Mariano Gálvez, Guatemala, Guatemala; 16 Escuela de Psicología, Pontificia Universidad Católica del Ecuador, Ambato, Ecuador; 17 Carrera de Psicología, Facultad de Filosofía, Universidad Nacional de Asunción, Asunción, Paraguay; 18 Escuela de Psicología, Facultad de Ciencias Sociales, Universidad Tecnológica de El Salvador, San Salvador, El Salvador; 19 Centro de Estudios Adlerianos, Instituto Alfred Adler Uruguay, Montevideo, Uruguay; 20 Programa de Psicología, Universidad de Ibagué, Ibagué, Colombia; 21 Servicio de Cirugía Maxilofacial, Hospital General Universitario Carlos Manuel de Céspedes, Universidad de Ciencias Médicas de Granma, Bayamo, Granma Cuba; 22 Colegio Estatal de Psicólogos en Intervención de Jalisco A.C. Guadalajara, Jalisco, México; 23 Departamento de Psicología, Universidad Católica San Pablo, Arequipa, Perú; 24 Lone Star College, Conroe, USA; 25 School of Health and Sport Sciences, Fundación Universitaria del Área Andina, Bogotá, Colombia; 26 Departamento de Psiquiatría, Facultad de Ciencias Médicas, Universidad Nacional de Asunción, San Lorenzo, Paraguay; 27 Asociación Nicaragüense para el desarrollo de la psicología – ANDEPSI, Managua, Nicaragua; 28 Universidad Jesús de Nazareth, San Pedro Sula, Honduras; 29 Instituto de Investigaciones Psicológicas, Facultad de Ciencias Sociales, Universidad de Costa Rica, San Pedro, Costa Rica; 30 Departamento de Investigación, Universidad Jesús de Nazareth, San Pedro Sula, Honduras; 31 Centro de Investigación y Asesoramiento Psicológico, Facultad de Humanidades, Comunicación y Artes, Universidad Privada de Santa Cruz de la Sierra, Santa Cruz, Bolivia; 32 Facultad de Ciencias de la Salud, Departamento de Enfermería, Universidad Católica del Maule, Talca, Chile; 33 Vicerrectorado de investigación, Escuela de Postgrado, Escuela de Medicina Humana, Universidad Católica de Santa María, Arequipa, Perú; 34 Carrera de Psicología, Facultad de Ciencias de la Salud, Universidad del Valle de Mexico, Ciudad de Mexico, Mexico; 35 Instituto de Investigación en Ciencias Sociales y Educación, Universidad de Atacama, Copiapó, Chile; 36 Asociación Panameña de Psicólogos, Ciudad de Panamá, Panamá; 37 Escuela de Psicología, Universidad Latina de Panamá, Ciudad de Panamá, Panamá; 38 Escuela de Psicologia, Universidad Latina de Costa Rica, San José, Costa Rica; 39 Departamento de Ciencias Sociales, Universidad de Los Lagos, Osorno, Chile

**Keywords:** Alignment method, COVID-19, COVID-SCORE-10, Invariance, Latin America

## Abstract

**Objectives:**

The present study aimed to evaluate the measurement invariance of a general measure of the perception of governmental responses to COVID-|19 (COVID-SCORE-10) in the general population of 13 Latin American countries.

**Methods:**

A total of 5780 individuals from 13 Latin American and Caribbean countries selected by non-probabilistic snowball sampling participated. A confirmatory factor analysis was performed and the alignment method was used to evaluate invariance. Additionally, a graded response model was used for the assessment of item characteristics.

**Results:**

The results indicate that there is approximate measurement invariance of the COVID-SCORE-10 among the participating countries. Furthermore, IRT results suggest that the COVID-SCORE-10 measures with good psychometric ability a broad spectrum of the construct assessed, especially around average levels. Comparison of COVID-SCORE-10 scores indicated that participants from Cuba, Uruguay and El Salvador had the most positive perceptions of government actions to address the pandemic. Thus, the underlying construct of perception of government actions was equivalent in all countries.

**Conclusion:**

The results show the importance of initially establishing the fundamental measurement properties and MI before inferring the cross-cultural universality of the construct to be measured.

**Supplementary Information:**

The online version contains supplementary material available at 10.1186/s41155-023-00277-9.

## Introduction

The COVID-19 pandemic has generated negative social, economic, educational and health consequences worldwide (Lazarus et al., 2020b). Since its emergence in China and until April 1, 2022, more than 480 million diagnosed cases of COVID-19 and more than 6 million deaths from the disease have been reported worldwide. The emergence and rapid spread of new SARS-CoV-2 virus variants, such as Delta and Omicron, means that the public health emergency will continue to be a public health emergency (Haque et al., 2022) and continue to exert pressure on governments around the world (White et al., 2021).

Since the beginning of the pandemic, the different governments of the world have implemented actions to contain the spread of the disease among their different populations (Lazarus et al., 2020b). These actions included quarantine, restrictions on the movement of people, and the closure of schools, places of worship, stores and industrial activities (Sebastiani et al., 2020). In addition, preventive behaviors such as the use of masks, hand washing, use of hand sanitizer, physical distancing and vaccination against COVID-19 have been promoted (Olapegba et al., 2021). It has even been recommended that preventive behaviors should be maintained after being fully vaccinated against COVID-19 (Aschwanden et al., 2021). However, compliance with these measures has varied among different environments (Sibley et al., 2020). It is possible that the lack of confidence in the government (Sibley et al., 2020) and the confusion generated by the unclear and contradictory information issued by some governmental sources (Gollust et al., 2020; Garrett, 2020) have generated the limited compliance or non-compliance with the aforementioned measures. Likewise, the pandemic may change people’s trust in government and institutions, where those faced with an external threat may have greater confidence in government and institutions because they have few additional options (Bavel et al., 2020).

During this pandemic, the leadership role of government is important, especially in a context of uncertainty about the effectiveness of the control measures in place (White et al., 2021). Greater trust in government authorities makes it more likely to comply with the recommended protective practices for dealing with the disease (Seale et al., 2020). This relationship has been observed previously during the H1N1 pandemic (Freimuth et al., 2014) and the Ebola epidemic (Blair et al., 2017). The different degrees of trust in government may be influenced by certain individual characteristics (Kavanagh et al., 2020). Thus, it is important to have a better understanding of people’s perceptions of government responses to the COVID-19 pandemic. People’s opinions are directed not only to the effectiveness of the measures implemented by governments, but also to other more specific actions, such as support for the most vulnerable groups (Lazarus et al., 2020b).

However, there are relatively few studies that have assessed people’s perceptions of the role of government during the pandemic, particularly in Latin America. The governments of Latin American countries face the pandemic in the midst of their own structural crises such as social inequality and poverty that have led to social and political polarization, in addition to a high prevalence of chronic diseases and a response with limited health resources (Ramírez de la Cruz et al., 2020). This has made Latin America and the Caribbean one of the regions of the world most affected by the pandemic (Anaya-Covarrubias et al., 2022). A recent study indicated that Latin America, along with Europe, is one of the regions that perceived their governments’ responses to COVID-19 as inadequate (Lazarus et al., 2020b). This negative perception leads to harsh criticism of the measures taken by the government (Paterlini, 2020).

A better understanding of perceptions of government responses to COVID-19 requires validated measures. To this end, the COVID-SCORE-10 (Lazarus et al., 2020a, b) was recently developed as a general measure of perceptions of government responses to COVID-19. Specifically, the COVID-SCORE-10 assesses people’s perceptions of socioeconomic support, continuity of health services, communication, and disease control measures. The COVID-SCORE-10 was developed from a longer version of 20 items, the COVID-SCORE-20 (Lazarus et al., 2020a). The choice of items for COVID-SCORE-10 was made by a panel of experts after a review of information on government responses to pandemics and other natural disasters presented above (Lazarus et al., 2020b). Initially, the COVID-SCORE-10 was developed in English and translated into different languages (such as Portuguese, Mandarin Chinese, French, German, Italian, Polish, Russian, Korean, Swedish, among others) under the assumption that it can obtain information from different countries and is sensitive to cultural differences. However, to our knowledge, there was no previous study that evaluated its measurement invariance (MI) across different countries and/or cultures.

Only the initial study reported that the measure was reliable (Cronbach’s alpha = 0.92) and unidimensional (Lazarus et al., 2020a, b). The latter was performed on the basis of a principal component analysis (PCA), which is part of the set of procedures known as Little Jiffy (Kaiser, 1960), which is the least recommended for assessing the internal structure of a measurement test (Lara & Soto, 2016). Regarding PCA, it is a method of reduction of observed variables or items, and not a factor analysis (Lloret-Segura et al., 2014), which takes into consideration the total variance (which includes the common and unique variance, in addition to the error variance) and leads to overestimate the factor loadings and distort the appropriate variance (Ferrando & Anguiano-Carrasco, 2010).

Conducting a cross-cultural study, similar to Lazarus et al. (2020a, b), should effectively address cultural influences on measure performance across countries (Ryan et al., 1999). However, most research comparing groups (including countries) does not assess the equivalence of the factor structure of the instruments between groups (Steinmetz et al., 2009). For this, the MI procedure makes it possible to evaluate whether an instrument works in the same way in all groups. Failure to establish MI would mean that the results of the comparison between groups may be erroneous and not replicable, since the differences between groups may not reflect true differences, but rather a different functioning of the instrument between the groups evaluated. This would mean that the theoretical and practical implications of cross-cultural studies may be limited or spurious (Nimon & Reio, 2011). In view of this, MI should be evaluated before drawing conclusions based on a group comparison (Jeong & Lee, 2019).

Although the COVID-SCORE-10 has been used in different countries, this does not mean that it can be used in different places without being certain that the concept and measurement of the perception of governmental actions against the COVID-19 is similar. In view of this situation, the present study aimed to evaluate the MI of the COVID-SCORE-10 in the general population of 13 Latin American countries. This will provide additional evidence for a general tool that can measure the perception of government actions on COVID-19 in various countries and that is sensitive to cultural differences, which would benefit researchers and public health policy makers. Having a measure that is invariant across countries will allow for understanding how people perceive their governments’ response to the COVID-19 pandemic in order to plan and adapt their public health interventions. This topic is considered a research priority (World Health Organization [WHO], 2020) and important for decision support for governments in Latin America and the Caribbean.

Additionally, once MI was tested, the performance and parameter estimates of individual COVID-SCORE-10 items were evaluated based on Item Response Theory (IRT). IRT models allow for a better understanding of the relationship between an individual’s responses to the COVID-SCORE-10 items with the underlying latent trait, in this case the perception of government responses to the COVID-19 (Embretson & Reise, 2000). IRT analysis has been suggested as an effective method for developing and optimizing the sensitivity of measurement instruments, and to our knowledge, there are no previous studies that have conducted item-level analyses of the COVID-SCORE-10 using IRT models.

## Method

### Participants

Participants were 5780 individuals from 13 countries in Latin America and the Caribbean (Argentina, Bolivia, Chile, Colombia, Cuba, Ecuador, El Salvador, Guatemala, Mexico, Paraguay, Peru, Uruguay, and Venezuela), who were selected by non-probability snowball sampling. Snowball sampling has been a common strategy used in studies during the COVID-19 pandemic due to the limitations for interaction between individuals (Leighton et al., 2021). We planned to recruit a minimum sample size of 200 individuals in each country, which is considered an adequate sample size for psychometric studies (Wilson Von Voorhis & Morgan, 2007). In addition, the number of participants in each country was also in line with the recommendations for confirmatory factor analysis and IRT models, which required minimum samples of 300 and 375, respectively (De Ayala, 1994; Tabachnick & Fidell, 2007). The number of participants in each country ranged from 322 (Peru) to 747 (El Salvador). To be part of the study, participants were of legal age and gave informed consent.

The sample showed a higher participation of women (*n* = 4093) as opposed to men (*n* = 1687). The mean (M) age of the total participants was 33.53 years (M = 29, IQR = 23–42), with Mexico having the youngest participants (M = 24.96, M = 21, IQR = 20–27) and Guatemala the oldest (M = 44.04 years, M = 42, IQR = 33–57). Most of the participants were single (61.23%) and with completed university studies (47.08%). In addition, almost 50% (52.56%) reported not having been diagnosed with COVID-19. Table 1 shows the sociodemographic characteristics of each country in greater detail.

**Table 1:** Sociodemographic information of the study sample

Variable	Argentina (*n* = 363)	Bolivia (*n* = 567)	Chile (*n* = 453)	Colombia (*n* = 462)	Cuba (*n* = 334)	Ecuador (*n* = 438)	El Salvador (*n* = 747)	Guatemala (*n* = 420)	Mexico (*n* = 484)	Paraguay (*n* = 417)	Peru (*n* = 322)	Uruguay (*n* = 393)	Venezuela (*n* = 386)
	n (%)	n (%)	n (%)	n (%)	n (%)	n (%)	n (%)	n (%)	n (%)	n (%)	n (%)	n (%)	n (%)
Gender													
Male	108 (29.8)	143 (25.2)	139 (30.7)	139 (30.1)	103 (30.8)	127 (29.0)	200 (26.8)	123 (29.3)	153 (31.6)	125 (30)	98 (30.4)	120 (30.5)	109 (28.2)
Female	255 (70.3)	421 (74.3)	314 (69.3)	322 (69.7)	231 (69.2)	311 (71.0)	546 (73.1)	297 (70.7)	331 (68.4)	292 (70)	224 (69.6)	273 (69.5)	276 (71.5)
Other	0 (0.0)	3 (0.5)	0 (0.0)	1 (0.2)	0 (0.0)	0 (0.0)	1 (0.1)	0 (0.0)	0 (0.0)	0 (0.0)	0 (0.0)	0 (0.0)	1 (0.3)
Marital status													
Single	198 (54.6)	247 (43.6)	264 (58.3)	368 (79.7)	194 (58.1)	289 (66)	502 (67.2)	172 (41.0)	407 (84.1)	258 (61.9)	251 (78)	227 (57.8)	166 (43.0)
Married	74 (20.4)	223 (39.3)	99 (21.9)	61 (13.2)	64 (19.2)	98 (22.4)	167 (22.4)	179 (42.6)	57 (11.8)	94 (22.5)	45 (14.0)	73 (18.6)	146 (37.8)
Cohabiting	45 (12.4)	31 (5.5)	61 (13.5)	23 (5.0)	65 (19.5)	22 (5.0)	40 (5.4)	31 (7.4)	7 (1.5)	45 (10.8)	21 (6.5)	63 (16.0)	22 (5.7)
Divorced	28 (7.7)	58 (10.2)	24 (5.3)	8 (1.7)	8 (2.4)	25 (5.7)	25 (3.4)	29 (6.9)	13 (2.7)	13 (3.1)	4 (1.2)	26 (6.6)	44 (11.4)
Widowed	18 (5.0)	8 (1.4)	5 (1.1)	2 (0.4)	3 (0.9)	4 (0.9)	13 (1.7)	9 (2.1)	0 (0.0)	7 (1.7)	1 (0.3)	4 (1.0)	8 (2.1)
Education													
University (complete)	159 (43.8)	418 (73.7)	266 (58.7)	120 (26.0)	167 (50.0)	189 (43.2)	170 (22.8)	267 (63.6)	128 (26.4)	269 (64.5)	135 (41.9)	176 (44.8)	260 (67.4)
University (incomplete)	137 (37.7)	85 (15)	106 (23.4)	127 (27.5)	152 (45.5)	140 (32.0)	262 (35.1)	98 (23.3)	276 (57.0)	112 (26.9)	105 (32.6)	127 (32.3)	73 (18.9)
Vocational school (complete)	17 (4.7)	42 (7.4)	43 (9.5)	53 (11.5)	7 (2.1)	11 (2.5)	31 (4.1)	19 (4.5)	35 (7.2)	7 (1.7)	29 (9.0)	26 (6.6)	19 (4.9)
Vocational school (incomplete)	2 (0.6)	3 (0.5)	6 (1.3)	3 (0.6)	2 (0.6)	5 (1.1)	2 (0.3)	0 (0.0)	1 (0.2)	1 (0.2)	7 (2.2)	4 (1.0)	1 (0.3)
High school (complete)	40 (11.0)	15 (2.6)	28 (6.2)	141 (30.5)	5 (1.5)	78 (17.8)	164 (22.0)	30 (7.1)	39 (8.1)	24 (5.8)	39 (12.1)	36 (9.2)	28 (7.3)
Incomplete high school or lower	8 (2.2)	4 (0.7)	4 (0.9)	18 (3.9)	1 (0.3)	15 (3.4)	118 (15.8)	6 (1.4)	5 (1.0)	4 (1.0)	7 (2.2)	24 (6.1)	5 (1.3)
Had COVID-19													
No	180 (49.6)	247 (43.6)	368 (81.2)	214 (46.3)	158 (47.3)	226 (51.6)	348 (46.6)	285 (67.9)	256 (52.9)	182 (43.6)	90 (28.0)	300 (76.3)	187 (48.4)
Most likely not	48 (13.2)	53 (9.3)	35 (7.7)	47 (10.2)	24 (7.2)	31 (7.1)	68 (9.1)	13 (3.1)	47 (9.7)	27 (6.5)	16 (5.0)	25 (6.4)	27 (7.0)
Most likely yes	25 (6.9)	78 (13.8)	6 (1.3)	73 (15.8)	55 (16.5)	59 (13.5)	181 (24.2)	25 (6.0)	46 (9.5)	56 (13.4)	68 (21.1)	7 (1.8)	59 (15.3)
Yes	110 (30.3)	189 (33.3)	44 (9.7)	128 (27.7)	97 (29.0)	122 (27.9)	150 (20.1)	97 (23.1)	135 (27.9)	152 (36.5)	148 (46.0)	61 (15.5)	113 (29.3)

### Instruments

#### Sociodemographic survey

The sociodemographic questionnaire was prepared for the purposes of this study and included questions about the participants’ sex, age, educational level, and having been diagnosed with COVID-19.

#### Global survey to assess public perceptions of government responses to COVID-19 (COVID-SCORE-10; Lazarus et al., 2020b)

The COVID-SCORE-10 is comprised of 10 items and aims to measure people’s perceptions of their government’s COVID-19 response actions. Each of the 10 items has five response options ranging from “strongly disagree = 1” to “strongly agree = 5”. For the scoring, a min–max transformation is applied to the sum of the items and multiplied by 100; in this way, the final scores are in the range between 0 and 100. The study used the Spanish version of White et al. (2021). The COVID-SCORE-10 items are (in parentheses and in italics the Spanish translation of each item):

1. The government helped me and my family meet our daily needs during the COVID-19 epidemic in terms of income, food, and shelter (*El gobierno nos ayudó a mí y a mi familia a satisfacer nuestras necesidades diarias durante la epidemia de la COVID-19 en términos de ingresos, alimentos y vivienda*)
2. The government communicated clearly to ensure that everyone had the information they needed to protect themselves and others from COVID-19, regardless of socioeconomic level, migrant status, ethnicity or language (*El gobierno se comunicó claramente para garantizar que todos tuvieran la información que necesitaban para protegerse a sí mismos y a otros de la COVID-19, independientemente de su nivel socioeconómico, estatus migratorio, origen étnico o idioma*)
3. I trusted the government’s reports on the spread of the epidemic and the statistics on the number of COVID-19 cases and deaths (*Confié en los informes del gobierno sobre la propagación de la epidemia y las estadísticas sobre el número de casos y muertes por COVID-19*)
4. The government had a strong pandemic preparedness team that included public health and medical experts to manage our national response to the COVID-19 epidemic (*El gobierno contaba con un sólido equipo de preparación para una pandemia que incluía expertos médicos y de salud pública para gestionar nuestra respuesta nacional a la epidemia de COVID-19*)
5. The government provided everyone with access to free, reliable COVID-19 testing if they had symptoms (*El gobierno brindó a todos acceso a pruebas de COVID-19 gratuitas y confiables si tenían síntomas*)
6. The government made sure we always had full access to the healthcare services we needed during the epidemic (*El gobierno se aseguró de que siempre tuviéramos pleno acceso a los servicios de atención médica que necesitábamos durante la epidemia*)
7. The government provided special protections to vulnerable groups at higher risk such as the elderly, the poor, migrants, prisoners and the homeless during the COVID-19 epidemic (*El gobierno brindó protecciones especiales a los grupos vulnerables con mayor riesgo, como los ancianos, los pobres, los migrantes, los prisioneros y las personas sin hogar, durante la epidemia de COVID-19*).
8. The government made sure that healthcare workers had the personal protective equipment they needed to protect them from COVID-19 at all times (*El gobierno se aseguró de que los trabajadores de la salud tuvieran el equipo de protección personal que necesitaban para protegerse del COVID-19 en todo momento*).
9. The government provided mental health services to help people suffering from loneliness, depression and anxiety caused by the COVID-19 epidemic (*El gobierno brindó servicios de salud mental para ayudar a las personas que sufren de soledad, depresión y ansiedad causadas por la epidemia de COVID-19*)
10. The government cooperated with other countries and international partners such as the World Health Organization (WHO) to fight the COVID-19 pandemic (*El gobierno cooperó con otros países y socios internacionales como la Organización Mundial de la Salud (OMS) para combatir la pandemia de COVID-19*).

### Procedure

The project was approved by the Institutional Committee for the Protection of Human Subjects in Research (CIPSHI) of the University of Puerto Rico (No. 2223-006) and informed consent to participate in this study was provided by the participants. However, the study followed the ethical guidelines of the American Psychological Association (APA, 2010) and the Declaration of Helsinki. All methods were carried out in accordance with relevant guidelines and regulations.

The study was conducted between September 15 and October 25, 2021 during the COVID-19 pandemic. An online survey was developed using Google Forms, which contained instructions for answering the survey, the study objectives, informed consent, and the COVID-SCORE-10 questions. The survey was distributed via social networks (Facebook, Instagram, and LinkedIn) and email. Participants were asked if they could disseminate the survey link to their personal contacts. This procedure was the same and was carried out simultaneously in the 13 Latin American and Caribbean countries that participated in the study. Responding to the survey was risk-free for the participants. Participants took part in the study on a completely voluntary basis and could discontinue their participation at any time. All participants gave informed consent to be part of the study. Participants were informed that their responses were completely anonymous and confidential. Responding to the survey took an average of approximately 10 min. In addition, to complete and submit the online survey, participants should not leave any questions unanswered.

### Data analysis

We began by analyzing some descriptive statistics at the item level. Specifically, the mean, standard deviation, skewness and kurtosis were calculated. Next, a confirmatory factor analysis (CFA) was performed using the robust maximum likelihood method (MLR; Yuan & Bentler, 2000). It should be noted that the instrument has 5 response options, so it is plausible to use it instead of more sophisticated methods such as weighted least squares means and variance adjusted (WLSMV; Rhemtulla et al., 2012). The reason for selecting MLR over WLSMV is that the alignment method used for the invariance analyses is based on the former. The model fit was judged with the following indices: comparative fit index (CFI), Tucker-Lewis index (TLI), root-mean-square error of approximation (RMSEA) y standardized root-mean-square residual (SRMR). To assess the fit of the model to the data, the following guidelines were considered: CFI > 0.95, TLI > 0.95, RMSEA < 0.06 y SRMR < 0.08 (Hu & Bentler, 1999).

For the evaluation of invariance, the alignment method was used, which is recommended when evaluating a large number of groups, as in the present case (Asparouhov & Muthén, 2014). The objective of this method is to reduce the lack of invariance as much as possible in order to perform an unbiased comparison of latent means. This methodology requires establishing a priori tolerance values for the parameters examined (factor loadings and intercepts). Following previous recommendations, conservative values were selected for both factor loadings (λ = 0.40) and intercepts (ν = 0.20) (Fischer & Karl, 2019). In addition, R^2^ were calculated for each parameter; values close to 1 suggest compliance with invariance. Finally, the total percentage of non-invariant parameters was also examined; values greater than 25% would indicate lack of invariance (Muthén & Asparouhov, 2014).

It should be noted that the aim of the alignment procedure is to estimate latent mean differences, and it was developed as an alternative to multi-group confirmatory factor analysis (MGCFA; Asparouhov & Muthén, 2014). Indeed, we only seek approximate (not exact) measurement invariance when applying the alignment method. Thus, lack of invariance under MGCFA is not incompatible with approximate alignment invariance. On the other hand, it is true that most applications of the alignment optimization use variations of the maximum likelihood estimator, and thus assume that the variables are continuous in nature (e.g. Marsh et al., 2018). While evidence suggests that it is safe to treat Likert-type items as continuous in single-group CFA (given that there are at least five response options; Rhemtulla et al., 2012), this may not hold for MGCFA (Temme, 2006). Furthermore, to the best of the authors’ knowledge, the robustness of this approach when using the alignment method has not been examined in simulation studies. Given the above, we decided to also conduct a MGCFA using the WLSMV estimator. Following recommendations for ordinal MGCFA, we first examined thresholds’ invariance, followed by the addition of factor loadings’ invariance (Temme, 2006; Wu & Estabrook, 2016). As expected, the results showed lack of (exact) measurement invariance (Supplementary Material 1). For transparency, we also make our dataset available for anyone interested in reproducing or improving our analyses. The database can be seen at the following link: https://osf.io/8ms6n.

Once the approximate invariance was verified, we proceeded with a graded response model (GRM) applied to the total sample. This model is part of the item response theory and consists of the estimation of two parameters (discrimination and difficulty) in polytomous items (Samejima, 2016). Specifically, one discrimination parameter (a) and k-1 difficulty parameters (b) are estimated for each item, where k is the number of response options. Discrimination refers to the ability of the item to distinguish between persons with high and low levels of the construct (θ). Difficulty parameters refer to the level of the construct (θ) at which the individual has a 50% probability of providing answers higher than indicated by parameter (Edelen & Reeve, 2007). With the information of both parameters, information curves were constructed for each of the items, which allow us to graphically examine the psychometric quality of the items in terms of reliability (Furr, 2018).

As mentioned, the alignment method used in the invariance analysis allows an unbiased comparison of the latent means between countries. In a complementary manner, this comparison was also made with the observed means. Although this procedure is methodologically inferior to the alignment method, it was applied to facilitate a simpler interpretation of the mean comparisons. Specifically, standardized mean differences were calculated with Cohen’s d index, which were interpreted considering the classic guide of 0.20, 0.50 and 0.80 as cut-off points for small, medium and large differences, respectively (Cohen, 1992).

The analyses were implemented in the R 4.0.3 program. For the CFA, the package lavaan 0.8–8 was used. For the alignment method, the sirt 3.9–4 package was used. Finally, the GRM was performed with mirt 1.33.2. The scripts used in this study can be seen at: https://osf.io/r5274.

## Results

### Preliminary analyses

Table 2 presents the descriptive statistics for each item of the COVID-SCORE. In general, people tended to show less acceptance of the item 1 (The government helped me and my family meet our daily needs during the COVID-19 epidemic in terms of income, food, and shelter), while item 10 had a greater acceptance (The government cooperated with other countries and international partners such as the World Health Organization (WHO) to fight the COVID-19 pandemic). As for the skewness and kurtosis values, most of them are within the range between -1 and -1, or very slightly outside this range.

**Table 2:** Item-level descriptive statistics of the COVID-SCORE

Country	Statistic	COVID-SCORE Items									
		1	2	3	4	5	6	7	8	9	10
Argentina (*n* = 363)	M	1.80	2.65	3.14	2.45	2.98	2.70	2.57	2.36	1.88	2.95
	SD	1.14	1.34	1.40	1.33	1.44	1.38	1.27	1.20	1.02	1.23
	g1	1.21	0.18	-0.24	0.51	-0.02	0.28	0.37	0.50	0.85	-0.17
	g2	0.36	-1.24	-1.22	-0.94	-1.36	-1.17	-0.90	-0.80	-0.27	-0.92
Bolivia (*n* = 567)	M	1.84	2.29	2.45	1.77	2.13	1.82	1.98	1.86	1.66	2.41
	SD	1.16	1.23	1.25	1.01	1.17	1.06	1.06	1.05	0.95	1.09
	g1	1.24	0.54	0.35	1.22	0.75	1.18	0.81	1.07	1.37	0.22
	g2	0.52	-0.85	-0.99	0.78	-0.42	0.61	-0.24	0.31	1.20	-0.78
Chile (*n* = 453)	M	2.36	2.67	2.67	2.21	3.08	2.64	2.67	2.47	1.83	3.09
	SD	1.29	1.36	1.37	1.28	1.37	1.31	1.33	1.24	1.03	1.09
	g1	0.48	0.24	0.25	0.75	-0.09	0.33	0.30	0.44	1.22	-0.30
	g2	-0.95	-1.24	-1.23	-0.58	-1.23	-1.00	-1.03	-0.82	0.86	-0.27
Colombia (*n* = 462)	M	1.86	2.60	2.79	2.18	2.51	2.32	2.49	2.46	2.18	2.87
	SD	1.19	1.31	1.30	1.15	1.28	1.20	1.21	1.24	1.14	1.12
	g1	1.18	0.26	0.09	0.59	0.27	0.50	0.28	0.31	0.61	-0.13
	g2	0.25	-1.07	-1.09	-0.61	-1.11	-0.76	-0.91	-1.00	-0.53	-0.60
Cuba (*n* = 334)	M	2.54	4.05	2.80	3.15	3.46	3.54	3.56	2.84	3.44	4.20
	SD	1.35	1.21	1.47	1.38	1.38	1.35	1.29	1.40	1.31	1.01
	g1	0.35	-1.27	0.13	-0.24	-0.47	-0.55	-0.62	0.13	-0.42	-1.31
	g2	-1.10	0.71	-1.38	-1.16	-1.06	-0.91	-0.63	-1.25	-0.89	1.38
Ecuador (*n* = 438)	M	1.98	2.67	2.61	2.28	2.43	2.45	2.51	2.50	2.41	2.93
	SD	1.31	1.33	1.29	1.26	1.35	1.32	1.27	1.28	1.26	1.23
	g1	1.05	0.23	0.29	0.64	0.49	0.42	0.37	0.40	0.48	-0.01
	g2	-0.22	-1.10	-0.94	-0.61	-0.94	-0.99	-0.86	-0.90	-0.78	-0.81
El Salvador (*n* = 747)	M	3.14	3.54	3.06	3.02	3.39	3.34	3.33	3.50	2.77	3.66
	SD	1.38	1.31	1.36	1.29	1.35	1.31	1.31	1.30	1.32	1.14
	g1	-0.20	-0.60	-0.12	-0.09	-0.42	-0.36	-0.32	-0.51	0.17	-0.56
	g2	-1.17	-0.71	-1.11	-0.98	-0.96	-0.92	-0.94	-0.78	-1.02	-0.42
Guatemala (*n* = 420)	M	1.73	2.36	2.30	1.76	2.18	1.95	1.96	1.94	1.65	2.28
	SD	1.19	1.38	1.31	1.08	1.32	1.14	1.17	1.14	0.99	1.19
	g1	1.50	0.57	0.58	1.42	0.77	1.01	1.06	1.12	1.47	0.49
	g2	1.06	-0.97	-0.89	1.33	-0.67	0.14	0.13	0.40	1.46	-0.74
Mexico (*n* = 484)	M	1.60	2.60	2.84	2.38	2.79	2.50	2.49	2.58	2.34	2.98
	SD	1.03	1.32	1.27	1.27	1.39	1.28	1.23	1.33	1.20	1.17
	g1	1.60	0.22	0.02	0.49	0.14	0.37	0.33	0.29	0.47	-0.05
	g2	1.59	-1.12	-1.03	-0.83	-1.22	-0.94	-0.83	-1.08	-0.75	-0.68
Paraguay (*n* = 417)	M	1.90	2.79	3.32	2.15	2.93	2.09	2.22	2.25	1.90	2.74
	SD	1.19	1.35	1.22	1.17	1.35	1.15	1.18	1.18	1.09	1.14
	g1	1.05	0.05	-0.45	0.65	-0.09	0.79	0.62	0.55	1.01	-0.05
	g2	-0.16	-1.25	-0.69	-0.64	-1.27	-0.31	-0.63	-0.76	0.11	-0.71
Peru (*n* = 322)	M	2.36	3.02	3.12	2.55	2.67	2.63	2.82	2.88	2.61	3.12
	SD	1.32	1.28	1.22	1.17	1.28	1.20	1.19	1.26	1.18	1.15
	g1	0.51	-0.14	-0.10	0.29	0.18	0.25	-0.02	0.03	0.21	-0.19
	g2	-0.99	-0.96	-0.79	-0.67	-1.05	-0.78	-0.86	-0.98	-0.80	-0.59
Uruguay (*n* = 393)	M	2.25	3.20	3.37	3.45	3.52	3.10	2.98	3.42	2.79	3.31
	SD	1.33	1.29	1.23	1.26	1.22	1.30	1.28	1.17	1.30	1.12
	g1	0.60	-0.20	-0.30	-0.46	-0.46	-0.05	0.02	-0.38	0.08	-0.33
	g2	-0.90	-0.99	-0.79	-0.74	-0.66	-1.02	-0.99	-0.57	-1.18	-0.33
Venezuela (*n* = 386)	M	1.31	2.06	1.66	1.56	1.67	1.58	1.61	1.66	1.37	1.96
	SD	0.83	1.29	1.11	1.02	1.10	1.02	1.01	1.06	0.81	1.21
	g1	3.15	0.88	1.61	1.86	1.62	1.78	1.69	1.61	2.53	1.04
	g2	9.80	-0.57	1.59	2.62	1.73	2.35	2.20	1.78	6.57	-0.02
Overall (*n* = 5786)	M	2.10	2.81	2.78	2.38	2.76	2.52	2.57	2.55	2.22	2.97
	SD	1.32	1.40	1.36	1.32	1.41	1.36	1.33	1.34	1.26	1.27
	g1	0.86	0.09	0.11	0.52	0.16	0.40	0.34	0.36	0.69	-0.08
	g2	-0.57	-1.27	-1.19	-0.91	-1.28	-1.05	-1.03	-1.07	-0.62	-0.92

*M* Mean, *SD* Standard Deviation, *g1* Skewness, *g2* Kurtosis

When proceeding with the CFA, an acceptable fit was observed in almost all countries (Table 3). The most notable exception was Uruguay, especially in relation to the TLI and RMSEA. In examining possible modifications, no conceptually defensible respecification was identified. Therefore, it was decided to continue with the initial model, even with the suboptimal fit for Uruguay. Table 3 also presents the factor loadings and internal consistency reliability. For the latter, values between 0.86 (Mexico) and 0.93 (Ecuador) were found, indicating adequate reliability.

**Table 3:** CFA’s fit indices, factor loadings and internal consistency reliability of the COVID-SCORE

Country	Fit Indices					Factor Loadings										ɑ	ω
	χ^2^	CFI	TLI	RMSEA	SRMR	1	2	3	4	5	6	7	8	9	10		
Argentina	73.81	.97	.97	.06	.03	.61	.75	.66	.78	.75	.87	.79	.76	.63	.74	.92	.92
Bolivia	113.78	.95	.94	.06	.04	.54	.68	.53	.63	.69	.85	.79	.82	.69	.53	.89	.89
Chile	134.30	.94	.92	.08	.04	.41	.69	.73	.77	.65	.80	.76	.74	.61	.58	.89	.90
Colombia	148.99	.93	.91	.08	.05	.62	.65	.55	.72	.71	.78	.80	.72	.78	.67	.90	.90
Cuba	73.74	.96	.95	.06	.04	.63	.54	.68	.70	.70	.77	.74	.73	.61	.55	.89	.89
Ecuador	142.03	.94	.93	.08	.04	.67	.65	.71	.80	.80	.86	.86	.79	.81	.69	.93	.93
El Salvador	122.02	.97	.96	.06	.03	.69	.73	.70	.70	.77	.80	.84	.80	.63	.67	.92	.92
Guatemala	128.68	.93	.91	.08	.05	.52	.63	.62	.68	.73	.85	.83	.80	.69	.71	.91	.91
Mexico	133.97	.94	.92	.08	.04	.46	.66	.56	.68	.67	.85	.78	.73	.68	.64	.89	.89
Paraguay	112.44	.93	.91	.07	.05	.47	.50	.38	.62	.57	.79	.83	.76	.65	.56	.86	.86
Peru	84.87	.95	.94	.07	.04	.47	.64	.59	.68	.75	.83	.84	.82	.75	.72	.91	.91
Uruguay	162.72	.92	.89	.10	.05	.53	.79	.71	.70	.70	.82	.81	.77	.64	.72	.91	.91
Venezuela	88.70	.96	.95	.06	.03	.53	.60	.71	.70	.83	.86	.88	.79	.76	.74	.92	.92
Overall	836.90	.96	.95	.06	.03	.61	.71	.63	.75	.75	.85	.84	.80	.73	.72	.92	.92

All degrees of freedom were 35, and all *p*s < .001

### Approximate measurement invariance

Table 4 presents the results of the approximate invariance analysis with the alignment method. As can be seen, in no case are there marked deviations concerning factor loadings. On the other hand, when intercept invariance is examined, it is not satisfied in 23.1% of the cases. It should be noted that this value is just below our a priori criterion (25%). Therefore, it is decided that the invariance between countries is approximately fulfilled, but this result should be taken with caution due to the closeness between the observed percentage of non-invariant intercepts and the pre-established maximum limit.

**Table 4:** Approximate measurement invariance of the COVID-SCORE using the alignment method

Parameters	Items	Countries													*R*^2^	%
Loadings	1	AR	BO	CL	CO	CU	EC	SV	GT	MX	PY	PE	UY	VE	.990	0.0
	2	AR	BO	CL	CO	CU	EC	SV	GT	MX	PY	PE	UY	VE		
	3	AR	BO	CL	CO	CU	EC	SV	GT	MX	PY	PE	UY	VE		
	4	AR	BO	CL	CO	CU	EC	SV	GT	MX	PY	PE	UY	VE		
	5	AR	BO	CL	CO	CU	EC	SV	GT	MX	PY	PE	UY	VE		
	6	AR	BO	CL	CO	CU	EC	SV	GT	MX	PY	PE	UY	VE		
	7	AR	BO	CL	CO	CU	EC	SV	GT	MX	PY	PE	UY	VE		
	8	AR	BO	CL	CO	CU	EC	SV	GT	MX	PY	PE	UY	VE		
	9	AR	BO	CL	CO	CU	EC	SV	GT	MX	PY	PE	UY	VE		
	10	AR	BO	CL	CO	CU	EC	SV	GT	MX	PY	PE	UY	VE		
Intercepts	1	(AR)	BO	(CL)	CO	(CU)	EC	(SV)	GT	(MX)	PY	PE	UY	(VE)	.993	23.1
	2	(AR)	BO	CL	CO	(CU)	EC	SV	GT	MX	(PY)	PE	UY	VE		
	3	AR	BO	(CL)	CO	(CU)	EC	(SV)	GT	MX	(PY)	PE	UY	(VE)		
	4	AR	BO	CL	CO	CU	EC	SV	GT	MX	PY	PE	(UY)	VE		
	5	AR	BO	(CL)	CO	CU	EC	SV	GT	MX	(PY)	(PE)	(UY)	VE		
	6	AR	BO	CL	CO	CU	EC	SV	GT	MX	PY	PE	UY	VE		
	7	AR	BO	CL	CO	CU	EC	SV	GT	MX	PY	PE	UY	VE		
	8	(AR)	BO	CL	CO	(CU)	EC	SV	GT	MX	PY	PE	(UY)	VE		
	9	(AR)	BO	(CL)	CO	(CU)	(EC)	SV	GT	(MX)	PY	(PE)	(UY)	VE		
	10	AR	BO	CL	CO	(CU)	EC	SV	GT	MX	PY	PE	UY	VE		
Factor means		0.00	-0.81	-0.06	-0.25	0.84	-0.22	0.69	-0.79	-0.16	-0.46	0.09	0.40	-1.14		

By maximizing the invariance in the data, the alignment method allows for the comparison of scores, which can be seen in the last row of Table 4.

### Graded response model

Next, a GRM was applied to the COVID-SCORE items. As shown in Table 5, the items with the least discrimination were 1 and 3, while the most informative was item 6. Regarding the difficulty parameters, it is observed that item 1 was the most “difficult”, since even average values of the construct (θ ≈ 0) were associated with a 50% probability of answering the lowest option, whereas values of 2 SD above the average were required to have 50% probability of answering the highest option. In all other cases, some variation in the spectrum of the construct covered was observed.

**Table 5:** Graded response model parameter estimates for the COVID-SCORE

Item	*a*	*b*_*1*_	*b*_*2*_	*b*_*3*_	*b*_*4*_
1	1.70	0.02	0.50	1.26	2.06
2	2.12	-0.80	-0.21	0.50	1.32
3	1.71	-0.89	-0.22	0.64	1.56
4	2.50	-0.40	0.18	0.93	1.62
5	2.46	-0.69	-0.12	0.52	1.25
6	3.83	-0.48	0.07	0.69	1.29
7	3.54	-0.56	0.01	0.71	1.35
8	2.91	-0.55	0.05	0.73	1.42
9	2.34	-0.28	0.33	1.13	1.84
10	2.18	-1.13	-0.52	0.52	1.38

**Fig. 1:**
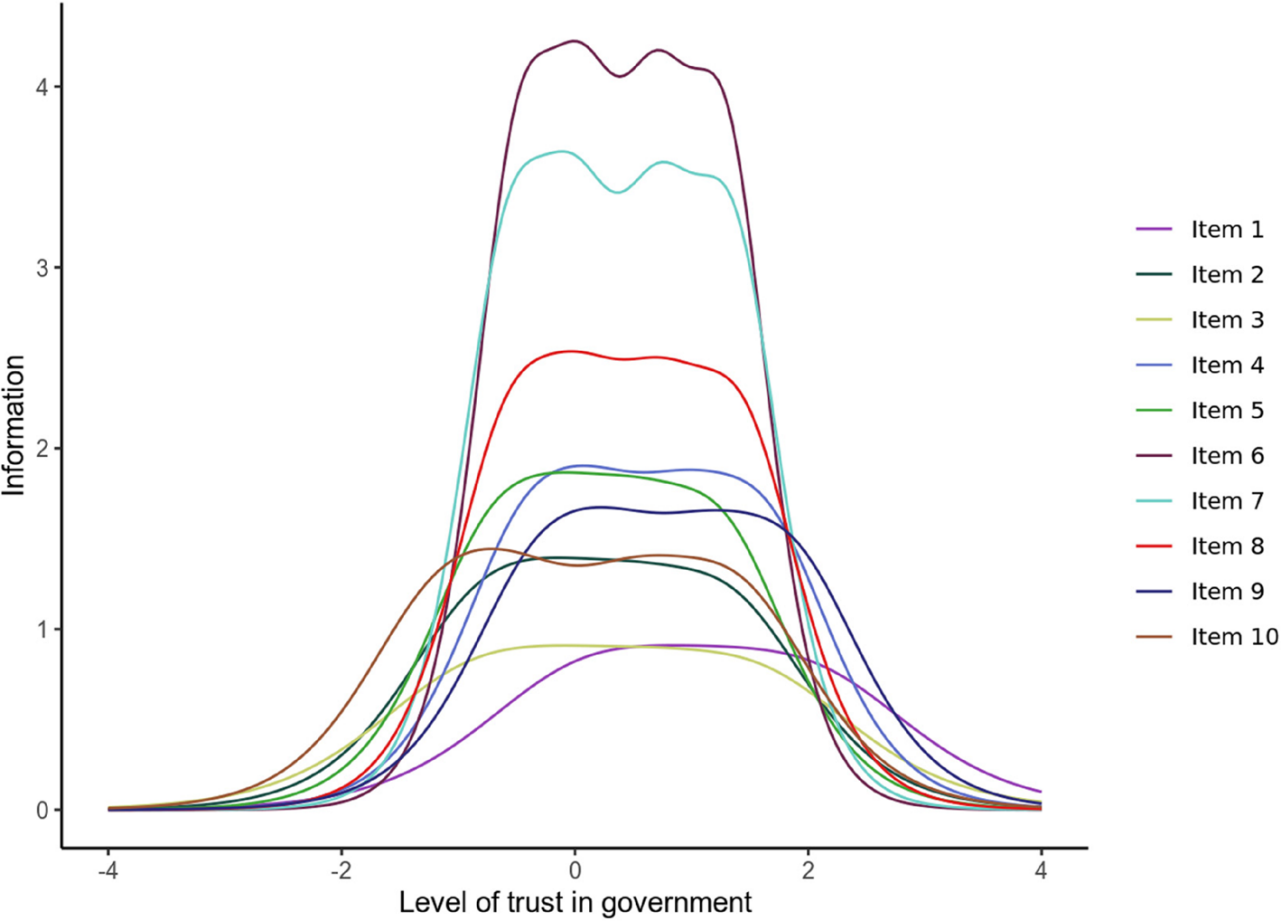
Item information curves of the COVID-SCORE

Based on the GRM parameters, information curves were constructed for each item of the COVID-SCORE (Fig. 1). These curves allow us to identify that items 6 and 7 are the most informative, especially at θ values close to the average. Taken together, the information curves demonstrate that the COVID-SCORE scores are more reliable at values close to the average of the latent variable.

### Mean comparison across countries

**Fig. 2:**
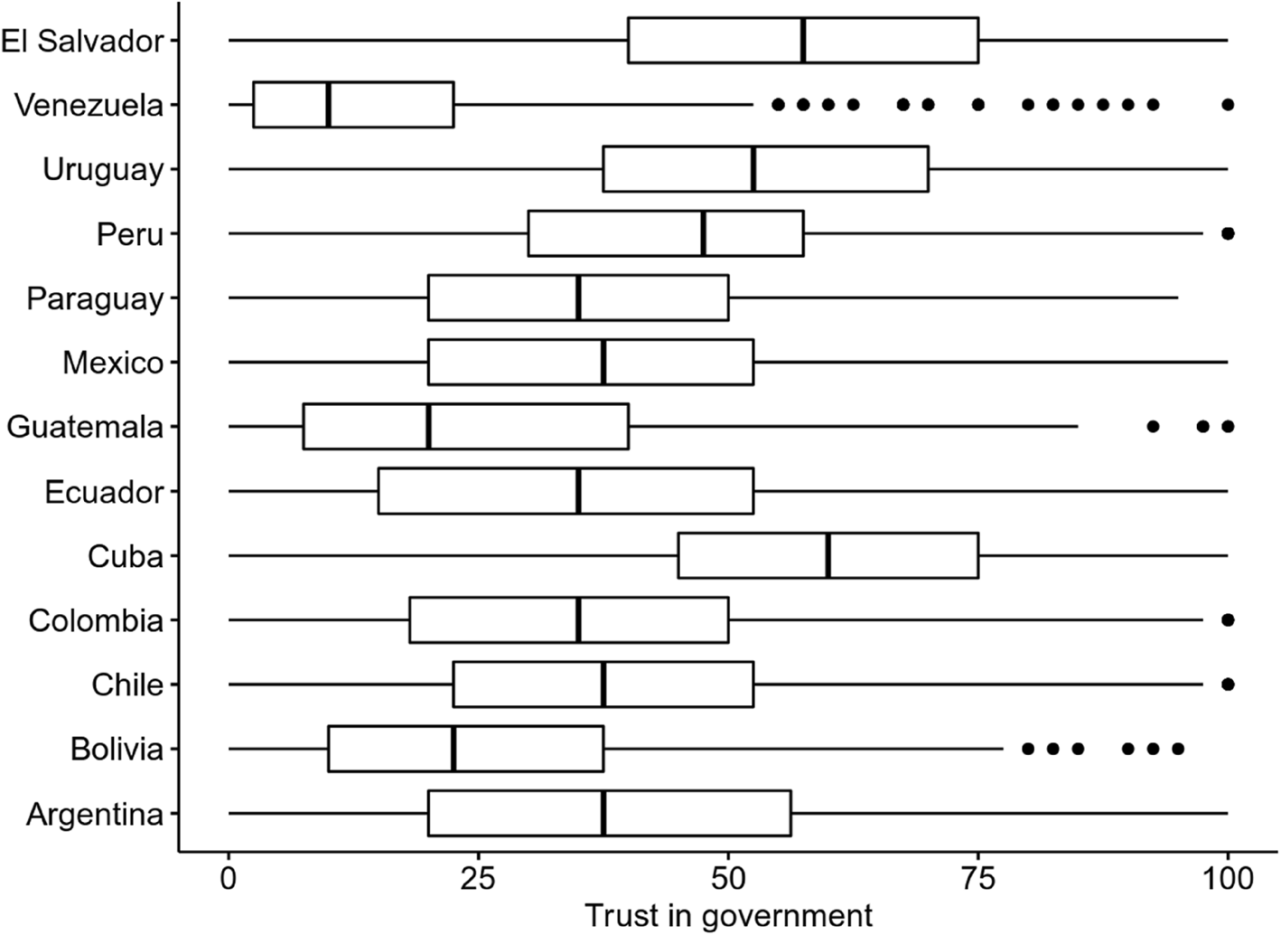
Boxplots comparing observed scores of the COVID-SCORE

When examining invariance with the alignment method, a comparison was already made between the latent measures of the variable perception of the actions carried out by the government. However, it was also decided to perform, in a complementary manner, a comparison between the transformed scores of the COVID-SCORE-10. In doing so, it was observed that the participants with the most positive perceptions of government actions to address the pandemic were Cuba, Uruguay and El Salvador (the differences between the three countries were small to negligible, *ds* < 0.50). On the other hand, the participants with the lowest positive perception were those from Venezuela, Guatemala and Bolivia (also *ds* < 0.50 among the three). Figure 2 shows boxplots that graphically represent these differences.

## Discussion

Due to the cross-cultural use of the COVID-SCORE-10 in different populations, the study aimed to assess whether the results are comparable between different countries by evaluating the MI of the scale. In particular, the cross-cultural replicability of the COVID-SCORE-10 was tested in 13 Spanish-speaking countries in Latin America and the Caribbean.

First, the evaluation of the factor structure of the COVID-SCORE-10 indicated the presence of a unidimensional model that fits the evaluated data well. In most countries, the CFI and TLI fit indices are above the cut-off value of 0.90 and the RMSEA and SRMR values are below 0.08, indicating an acceptable fit. It should be remembered that, the original COVID-SCORE-10 study only evaluated its factor structure by means of an exploratory factor analysis, but using procedures such as PCA, which have already been mentioned are inadequate as they are not a factor analysis method (Lloret-Segura et al., 2014). Only in Uruguay the values of some fit indices, such as TLI (0.89) and RMSEA (0.10) are slightly outside the range considered acceptable. The evaluation of a model with correlated errors could have improved model fit in all countries, including Uruguay; however, it has been suggested that this procedure could overestimate or underestimate reliability due to the presence of variance unrelated to the construct and thus generate a bias in the interpretation of COVID-SCORE-10 accuracy (Yang & Green, 2010).

On the other hand, the findings showed that the COVID-SCORE-10 is highly reliable in the 13 participating countries. In this sense, the COVID-SCORE-10 is likely to have balanced and easy-to-understand questions, resulting in consistent responses from individuals and generating good reliability.

On the other hand, although the COVID-SCORE-10 could be successfully replicated in each country independently, this is the first study to analyze its cross-national MI. Thus, identifying the generalizability of COVID-SCORE-10 scores is important for comparing groups internationally (Odell et al., 2021). The findings from the approximate invariance alignment approach indicate that the non-invariance for factor loadings (0%) and intercepts (23.1%) were within the recommended 25% limit (Muthén & Asparouhov, 2014), which provides greater reliability in the invariance results. In this sense, the alignment method indicated that no factor loadings challenged invariance.

On the other hand, if the non-invariance results had exceeded the 25% limit, a Monte Carlo simulation study would be needed to specifically identify the sources of non-invariance (Muthén & Asparouhov, 2014). Thus, the presence of an acceptable approximate invariance in the COVID-SCORE-10 was suggested to support its use for an unbiased comparison of the average levels of perception of government actions against the COVID-19 among the 13 countries assessed. Therefore, it can be suggested that the underlying construct of perception of government actions, as measured by the COVID-SCORE-10, was equivalent across countries. However, this result should be taken with caution due to the closeness between the observed percentage of non-invariant intercepts and the pre-established maximum limit.

The findings on invariance also provide further evidence to consider the alignment method as a suitable strategy for testing MI when the number of groups is large, which is difficult to achieve with the traditional approach based on CFA. Also, the alignment method allows estimating and comparing latent means despite partially invariant measurements (Cieciuch et al., 2018), which, in turn, automates and simplifies comparative analyses. Due to the ability of the alignment method to work with several groups, it is possible to test MI in different subpopulations within countries (Munck et al., 2018).

After testing for invariance, COVID-SCORE-10 scores were compared. It was observed that participants from Cuba, Uruguay and El Salvador had the most positive perceptions of government actions to address the pandemic. In the case of Cuba, the government, 1 month before the first case of COVID-19 was detected in its territory, created the Cuban Scientific Group for the Confrontation of COVID-19, which has been important in making decisions to control the pandemic (Castellanos-Serra, 2020; Díaz-Canel Bermúdez & Núñez Jover, 2020). This allowed immunological strategies to be applied during the outbreak of COVID-19 in Cuba, such as the development and application of an antibody detection test, the application of immunotherapeutics developed in Cuba to patients with COVID-19, and the application of a new antibody detection test (Pereda et al., 2020; Venegas Rodríguez et al., 2020) and the implementation of preventive strategies for vulnerable populations with products developed in Cuba for the immune system (Castellanos-Serra, 2020).

Uruguay is considered to be one of the most successful cases in the region in containing COVID-19, without implementing a general suppression strategy (González-Bustamante, 2021). The success of the initial containment of the pandemic by the Uruguayan government would lie, in part, in the rapid declaration of a state of health emergency throughout the country upon detection of the first cases and the closure of borders, schools and other activities that caused crowding. In addition, they appealed to personal responsibility to control the spread of the virus through voluntary self-confinement, but without mandatory blocking (e.g., no restrictions on meetings or public transportation, and no very lax quarantine) and seeking support from their scientific community to increase their testing capacity (e.g., no restrictions on public transportation, and no very lax quarantine) (Moreno et al., 2020).

Regarding this last point, it has been suggested that the evidence-based policies adopted by the Uruguayan government, together with a strong public health system and scientific innovations, are some of the main factors of success. For the development of evidence-based policies, scientific, medical-epidemiological, economic and educational aspects have been considered by a scientific advisory group made up of important figures in the government that provided recommendations on the different responses to the pandemic and the economic reactivation of Uruguay (Pittaluga & Deana, 2020). In this regard, the government of Uruguay developed a balanced strategy that allowed containing the social consequences of the pandemic and maintaining some degree of economic activity (Azerrat et al., 2021). In El Salvador, a few days after the first case of COVID-19 was detected, a strict containment was implemented, closing public transportation, schools and all stores, except those selling essential foodstuffs.

To try to mitigate the economic impact, the Salvadoran government made cash transfers of US$300 to workers in the informal sector; in addition, utility and loan payments were frozen, and millions of food baskets were distributed (Lagarde et al., 2020). In addition, El Salvador has been one of the Central American countries that have reached a proportion of direct beneficiaries, due to the fact that it has the three main components of the social protection information systems (social registry, single registry of beneficiaries and interoperability) (Cejudo et al., 2020). The actions taken in Cuba, Uruguay and El Salvador have been able to positively affect Cubans’ perception of their government’s actions to address COVID. In addition, countries such as Uruguay and Cuba are among those with lower income inequalities, smaller poverty gaps, higher per capita spending and higher public spending on health, which leads to better health outcomes (Giovanella et al., 2020).

However, it has also been reported that Venezuela, Guatemala and Bolivia presented the lowest positive perception of government actions. In the case of Venezuela, COVID-19 has been a serious threat that adds to the daily struggle of people to obtain basic foodstuffs in the midst of a political and economic crisis (Cooper, 2020). In this sense, the Venezuelan government’s response cannot be isolated from the country’s political situation. Therefore, in parallel, the country has had to face the pandemic and consolidate its control over a questioned political life (Østebø, 2020). Although official reports revealed that the country had some of the lowest incidence rates of COVID-19 in Latin America, it is possible that these figures are erroneous and therefore higher; in addition, the country did not have a standardized treatment process and used diagnostic tests with high false negative rates, which were rare (Bates et al., 2021). In addition, due to the fact that the Venezuelan government of President Maduro is not recognized by several countries, including the United States and the European Union, there have been problems for the arrival of vaccine against COVID-19, depending on Russia and China for the distribution of vaccines (Andrade, 2021).

In Guatemala, it has been suggested that the measures that, in other countries, were effective in controlling the pandemic, exacerbated the inequities present in the country and expressed the absence of social protection for citizens (Caridad et al., 2020). This, coupled with a weak and precarious health system to address the health crisis, lack of water, low coverage and malfunctioning of hospitals and lack of medicines, have led Guatemalans to perceive that their government had problems in dealing with the Covid-19 pandemic (Guillén & Pérez, 2021).

As in other countries, in Bolivia, the high percentage of informality and the persistent inequity in health benefits have amplified the impact of the pandemic and explain the poor results in containing the pandemic in the country (Hummel et al., 2021). In the same way, the political crisis over the legitimacy of the government meant that Bolivia did not have the conditions to carry out a coordinated response, with different results throughout the country (Velasco-Guachalla et al., 2021). Moreover, in Bolivia, citizen support for governmental measures has been lower, due to weaknesses in leadership and in the commitment to protect the life and health of the people, with a tendency to favor the economic interests of the elites (Giovanella et al., 2020). These adverse conditions did not contribute to a better perception of governmental actions to address the pandemic.

In general, in all Latin American countries, heterogeneity in the development of the epidemic, state capacity and pressure on health systems are significant factors for the rapid implementation of pandemic control strategies (Gallegos et al., 2020). Latin American countries have been challenged to respond to COVID-19 despite low budgetary support. Thus, the appropriate formulation of public health policies together with effective national diagnostic and vaccination strategies have been and will be key to the management of the pandemic, the reactivation of the economy and the alleviation of the poverty generated by the pandemic.

Additionally, the performance of the COVID-SCORE-10 items was evaluated based on the IRT. It was observed that item 6 is one of the most informative on the perception of government actions and refers to ensuring access to health services. As mentioned earlier, wide inequalities in effective access to health services are common in Latin America (Garcia et al., 2020), so it is not surprising that item 6 is one of the items that can provide the most information on the actions of Latin American governments to address the pandemic. Likewise, item 7, referring to the protection of the most vulnerable groups, is another item that allows us to obtain more information on the perception of the government’s actions. In this sense, many Latin American governments have seen the need to mitigate the effects of the pandemic on vulnerable groups through social pension programs and economic transfers to families, which function mainly as redistributive and social investment measures (Barrientos, 2020). However, these programs have been insufficient to compensate for the lack of work and income for the poorest segments of the population and for those informal workers at high risk of poverty (Busso et al., 2021).

In general, the impact on the most vulnerable groups of the population, limited access to social welfare and health services are the main concerns of the population in Latin America and the Caribbean (Benítez et al., 2020). Overall, the IRT results suggest that the COVID-SCORE-10 measures with good psychometric ability a broad spectrum of the construct, especially around average levels. That is, the instrument reliably measures trust in government for most people.

Despite the diversity of countries and consistent results, the present study has limitations that should be taken into account when interpreting the results. First, due to government regulations to prevent and control the COVID-19 pandemic, snowball sampling was adopted. This may generate the presence of partial bias and not have representative samples in each country. In added, the snowball sampling did not allow for adequate gender balance, with the majority of participants being female. Similarly, most had completed university education. This may have generated another bias, as less educated participants tended to have probably less access to the Internet, which was imperative due to the online nature of the study. Likewise, although we wanted to have the largest possible participation of countries, most of them came from South American countries, so there may be some type of regional bias in the findings. In view of the above, further studies should use more balanced sampling methods to allow greater generalization of the findings. Another limitation was that the data were collected using self-report methods.

Therefore, it is possible that the responses were affected by recall bias or social desirability. On the other hand, although a comparison was made between COVID-SCORE-10 scores across countries, the study alone cannot provide an exploration of how the economic and sociodemographic conditions of the participating countries affect individuals’ perceptions of government actions to address COVID-19. Thus, future studies should address the issue more directly by evaluating, for example, countries with very different socioeconomic characteristics. In addition, a longitudinal study design could be more informative of the evolution of individuals’ perceptions of government actions to address COVID-19 over time, beyond a single comparison.

## Conclusion

In conclusion, the study contributes to the knowledge of the factor structure of the COVID-SCORE-10 in different populations by presenting the MI results and characteristics of the scale items in 13 Latin American and Caribbean countries. The results show the importance of initially establishing the fundamental measurement properties and MI before inferring the cross-cultural universality of the construct to be measured. In sum, the findings provide evidence to test the external validity and cross-cultural applicability of the conceptualization and operationalization of the perception of government actions vis-à-vis COVID-19. Generalizability is an important characteristic when evaluating any measurement instrument. They also provide scholars and practitioners with strong evidence of cross-cultural variations in perceptions of how different governments have dealt with the pandemic. There is relatively little research on the perception of government actions vis-à-vis COVID-19 in the LAC region. In this sense, the availability of a psychometrically sound and invariant measure of the perception of government actions could motivate researchers to include the LAC region in cross-cultural research on the topic. However, future studies should provide more complete data on the evidence for the validity of COVID-SCORE-10 as well as an assessment of the impact of culture on perceptions of government actions to address the pandemic.

### Supplementary Information

**Additional file 1.** Multi-group Confirmatory Factor Analysis of the COVID-SCORE-10 using the WLSMV Estimator.

## Data Availability

The data presented in this study are available on request from the corresponding author.

## Abbreviations

MLR: Robust maximum likelihood method
WLSMV: Weighted least squares means and variance adjusted
CFI: Comparative fit index
TLI: Tucker-Lewis index
RMSEA: Root-mean-square error of approximation
SRMR: Standardized root-mean-square residual
GRM: Graded response model
CFA: Confirmatory factor analysis
MI: Measurement invariance
PCA: Principal component analysis
IRT: Item Response Theory
WHO: World Health Organization
