# Supplementary material for: Cross-cultural invariance of the Spanish version of the COVID-19 Assessment Scorecard to measure the perception of government actions against COVID-19 in Latin America
Source: Psicol Reflex Crit. 2023 Nov 8;36:34. doi: 10.1186/s41155-023-00277-9 (PMC10632328; doi:10.1186/s41155-023-00277-9)
Supplement: Supplementary file 1 — Additional file 1. Multi-group Confirmatory Factor Analysis of the COVID-SCORE-10 using the WLSMV Estimator. [file 41155_2023_277_MOESM1_ESM.docx]

**Supplementary Material 1**

*Multi-group Confirmatory Factor Analysis of the COVID-SCORE-10 using the WLSMV Estimator*

| Model | χ2 | *gl* | *p* | CFI | TLI | RMSEA | Δχ2 | Δ*gl* | *p* |
| --- | --- | --- | --- | --- | --- | --- | --- | --- | --- |
| 1. Baseline (configural) | 2852.12 | 455 | <.001 | .97 | .96 | .11 |  |  |  |
| 2. Equal thresholds | 3366.18 | 695 | <.001 | .97 | .97 | .09 | 310.85 | 240 | .001 |
| 3. Equal thresholds and factor loadings | 3318.12 | 803 | <.001 | .97 | .98 | .08 | 176.62 | 108 | <.001 |
